# Supplementary material for: Breath Insights: Advancing Lung Cancer Early-Stage Detection Through AI Algorithms in Non-Invasive VOC Profiling Trials
Source: Cancers (Basel). 2025 May 16;17(10):1685. doi: 10.3390/cancers17101685 (PMC12110429; doi:10.3390/cancers17101685)
Supplement: Supplementary file 1 [file cancers-17-01685-s001.zip › cancers-3618787-supplementary.docx]

Supporting Information

**Breath Insights: Advancing Lung Cancer Early-Stage Detection Through AI Algorithms in Non-Invasive VOC Profiling Trials**

Bernardo S. Raimundo,^a^ Pedro M. Leitão,^b^ Manuel Vinhas,^b^ Maria V. Pires,^a^ Laura B. Quintas,^a^ Catarina Carvalheiro,^a^ Rita Barata,^a^ Joana Ip,^c^ Ricardo Coelho,^a^ Sofia Granadeiro,^a^ Tânia S. Simões,^a^ João Gonçalves,^a^ Renato Baião,^a^ Carla Rocha,^a^ Sandra Alves,^d^ Paulo Fidalgo,^e^ Alípio Araújo,^e^ Cláudia Matos,^a^ Susana Simões,^a^ Paula Alves,^f^ Patrícia Garrido,^a^ Marcos Pantarotto,^a^ Luís Carreiro,^a^ Rogério Matos,^a^ Cristina Bárbara,^f^ Jorge Cruz,^a^ Nuno Gil,^a^ Fernando Luis-Ferreira,^b*^ Pedro D. Vaz^a*^

^a^ Unidade de Pulmão, Centro Clínico Champalimaud, Fundação Champalimaud, Lisboa, Portugal

^b^ Departamento de Engenharia Electrotécnica e de Computadores, Faculdade de Ciências e Tecnologia, Universidade Nova de Lisboa, Quinta da Torre, Caparica, Portugal

^c^ Serviço de Radiologia, Centro Clínico Champalimaud, Fundação Champalimaud, Lisboa, Portugal

^d^ Unidade de Ensaios Clínicos, Centro Clínico Champalimaud, Fundação Champalimaud, Lisboa, Portugal

^e^ Unidade de Risco e Diagnóstico Precoce, Centro Clínico Champalimaud, Fundação Champalimaud, Lisboa, Portugal

^f^ Serviço de Pneumologia, Centro Hospitalar e Universitário Lisboa Norte, Lisboa, Portugal

**Corresponding Authors**

Dr. Fernando Luís-Ferreira

Departamento de Engenharia Electrotécnica e de Computadores, Faculdade de Ciências e Tecnologia, Universidade Nova de Lisboa, Quinta da Torre, 2829-516 Caparica, Portugal

Telephone: +351 212 949 631

E-mail: flf@fct.unl.pt

Dr Pedro D. Vaz

Unidade de Pulmão, Centro Clínico Champalimaud, Fundação Champalimaud, Av. Brasília, P-1400-038 Lisboa, Lisboa, Portugal

Telephone: +351 210 480 048

E-mail: pedro.vaz@fundacaochampalimaud.pt

**Volunteer recruitment & eligibility criteria**

The population was recruited between July 2020 and December 2023 comprising the following two groups: (1) LC patients, and (2) control (C) group of healthy subjects. Patients eligible for recruitment who fulfilled the eligibility criteria were referred by the clinical staff through the Multidisciplinary Team meetings and recruited. Healthy subjects were recruited from the hospital staff or externally. The latter were either relatives from the recruited patients or from the general public that offered themselves as volunteers.

The eligibility criteria at the time of enrollment were:

- LC group: (1) eighteen (18) years old or older; (2) able to understand language spoken and/or written; (3) able to provide consent based on previous information; (4) diagnosed with LC; (5) no previous or present diagnosis of another primary tumor at another location in the last five years.
- C group: (1) eighteen (18) years old or older; (2) able to understand language spoken and/or written; (3) able to provide consent based on previous information; (4) without infectious or inflammatory diseases; (5) no previous or present diagnosis of neoplasia.

Once recruited, LC patients and healthy controls were validated by the medical staff at the clinical site.

**Lung cancer histology**

The histology found for all LC patients is stripped down in Table S1.

**Table S1.** Full histology breakdown of all LC patients enrolled in the study.

| **Parameter** |  | **Lung Cancer Patients** |
| --- | --- | --- |
| Lung cancer type, *n* (%) |  |  |
|  | Adenocarcinoma | 49 (67.1) |
|  | Squamous cell | 6 (8.2) |
|  | Small cell | 5(6.9) |
|  | Other | 13 (17.8) |
|  | Carcinoid | 2 |
|  | Minimally invasive | 3 |
|  | Pleomorphic carcinoma | 1 |
|  | *In situ* carcinoma | 3 |
|  | Not otherwise specified | 4 |

**Assessment of breath sample collection easiness by recruited individuals**

During the sample collection procedure, a member of the research team was present assisting and monitoring the volunteers during their breath test. At the end of each test all volunteers were asked about the easiness of conducting the breath test. They were given three choices – Difficult, Easy and Very easy.

According to Figure S1 below a striking 99% of all enrolled volunteers across both groups, found the test easy or very easy.


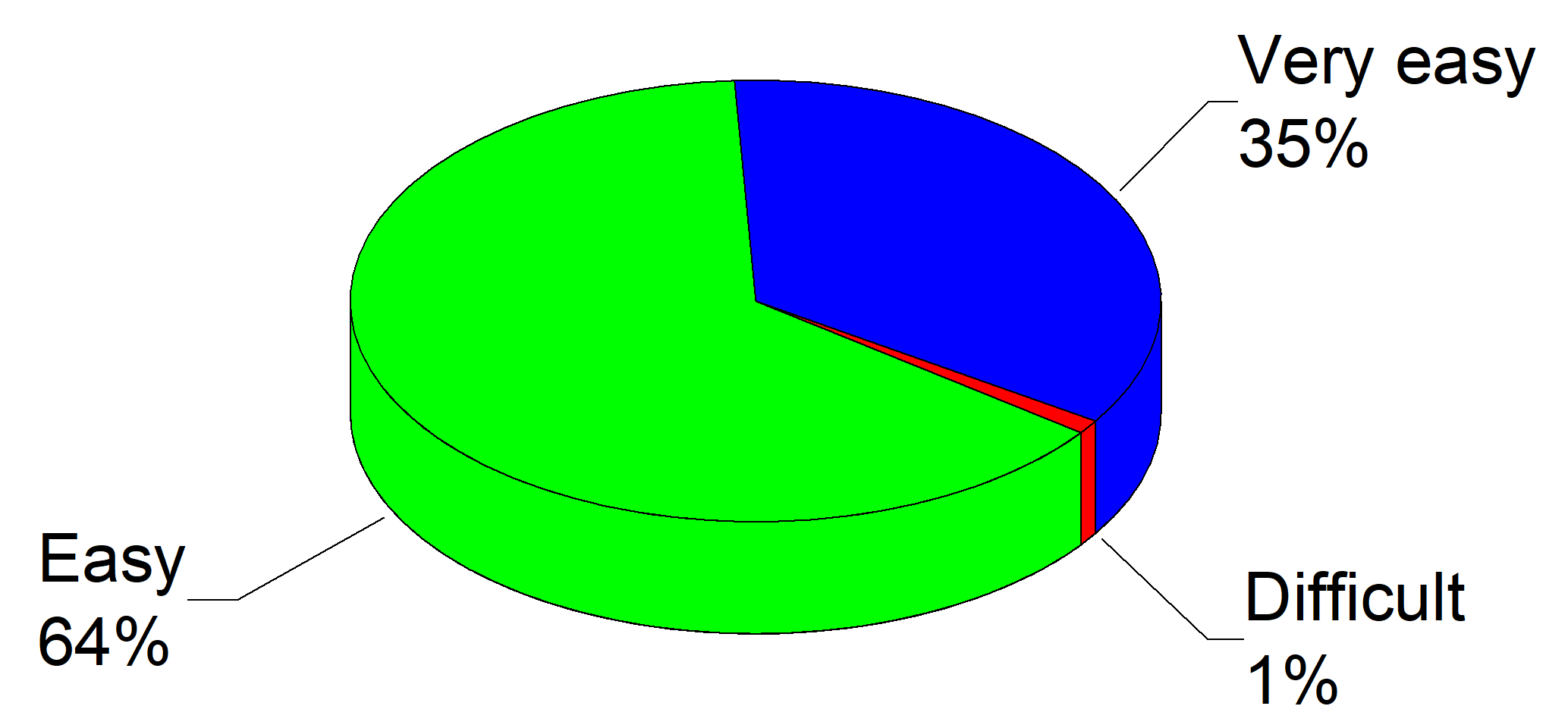


**Figure S1.** Results from the enquiry to volunteers.

**Classification methodology**

*Data processing*

Because each volunteer provided a set of four replicate profiles they were used as such. Hence, the total of 196 volunteers (123 Controls and 73 LC patients) yielded 784 profiles (492 control profiles and 292 LC profiles). For this work, two datasets of similar composition were analyzed (Table S2), with information relating to the analysis of volatile organic compounds in exhaled air samples from healthy patients and patients with Lung Cancer. We will refer to them as *'C vs LC all stages'*, and *'C vs LC stage IA'*. Figure S2 shows the representation for the first dataset, with the structure being the same for both.

**Table S2.** Dataset’s composition with percentages.

| Dataset | Control group | Lung cancer group | Total |
| --- | --- | --- | --- |
| 'C vs LC all stages' | 492 (63%) | 292 (37%) | 784 |
| 'C vs LC stage IA' | 492 (79%) | 128(21%) | 620 |

In order to be able to use this data in the various machine learning classifiers, it is necessary that in each row we have a sample, represented by C001, C002, etc., and in the columns the class and the values corresponding to the sampled retention time across the chromatogram (VOC values), which will provide us with the overall unique features useful for classification. The first dataset studied was the product of the combination of two datasets of similar constitutions, from control and LC cohorts, having proceeded to concatenate both with all the samples collected. Moreover, all columns with null values were eliminated, reducing the final dataset size.

From this point forward, no more preprocessing steps have been done for the classification algorithms.


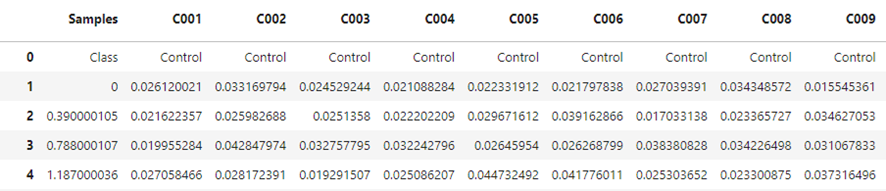


**Figure S2.** Original matrix representation of dataset 'C vs LC all stages' from raw data

Since we are faced with a dichotomy of classes, those were coded so that the *'C'* class was represented by *'0'* and the *'LC'* class by *'1'*, maintaining this designation for the remainder of the work and respective analysis of results. This operation was done in both datasets (Figure S3).


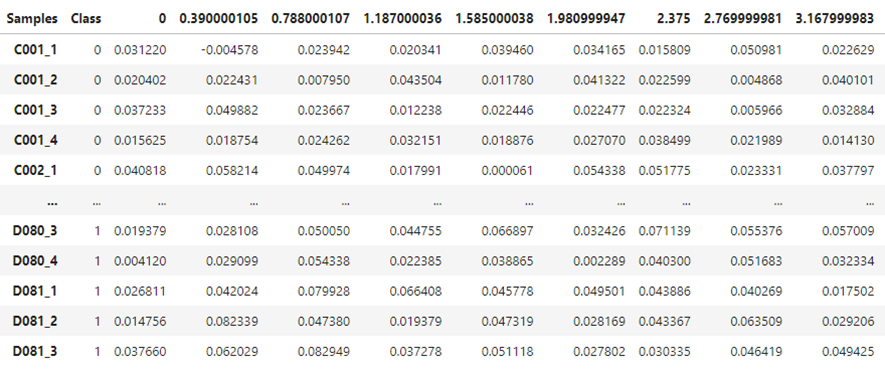


**Figure S3.** Matrix representation for dataset *'Control vs LC'* after class reorganization.

**Machine Learning Analysis**

*Machine learning – Unsupervised learning*

By using unsupervised learning, the aim was to understand whether it would be possible to draw some type of conclusions by visualizing the data and observing possible groups that reflected an important set of characteristics for our classification problem. Two algorithms were tested, K-Means Clustering (KMC, Figures S4 and S5) and Principal Components Analysis (PCA, Figures S6 and S7), using the datasets *'C vs LC all stages'* and *'C vs LC stage IA'*. Neither algorithm was able to yield a classification of the classes, mainly due to the large size of the data, thus not allowing us to draw any conclusions using these models.

| 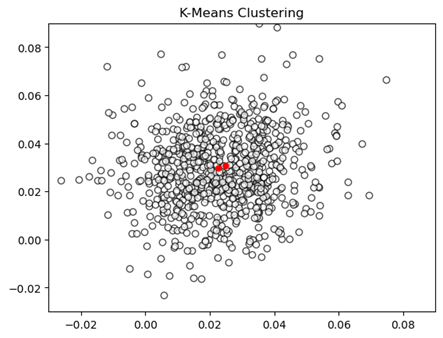 | 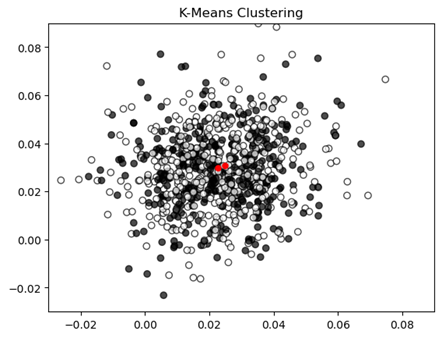 |
| --- | --- |

**Figure S4.** K-Means Clustering results without (right) and with (left) class discrimination for dataset 'C vs LC all stages'.

| 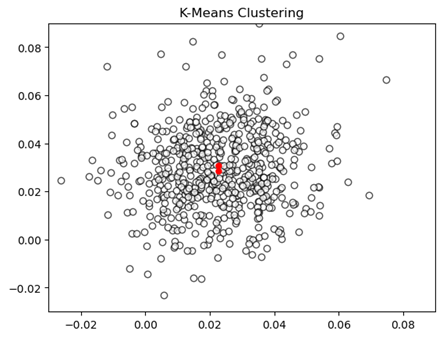 | 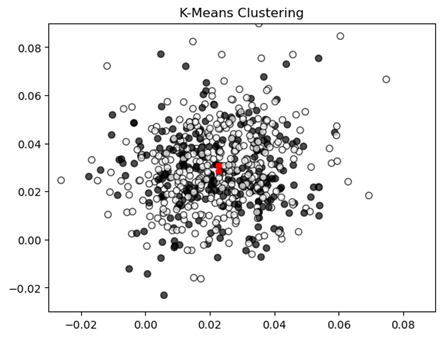 |
| --- | --- |

**Figure S5.** K-Means Clustering results without (right) and with (left) class discrimination for dataset 'Control vs LC stage IA'.

| 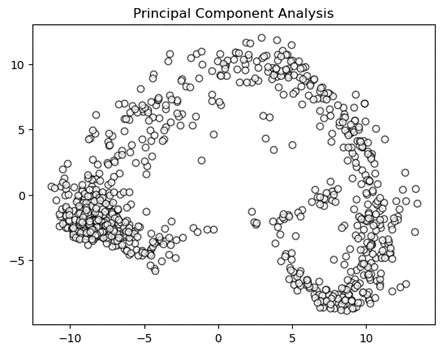 | 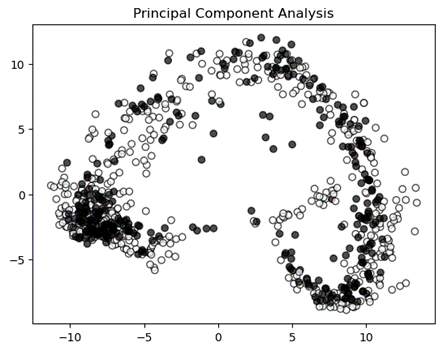 |
| --- | --- |

**Figure S6.** Principal Component Analysis results without (right) and with (left) class discrimination for dataset *'C vs LC all stages'*.

| 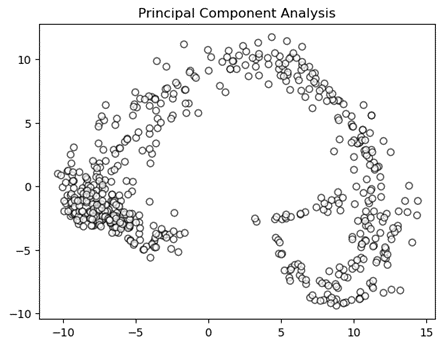 | 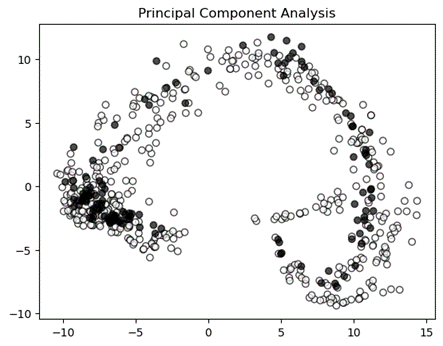 |
| --- | --- |

**Figure S7.** Principal Component Analysis results analysis without (right) and with (left) class discrimination for dataset *'C vs LC stage IA'*

*Machine learning – supervised learning*

The models used were the following – Decision Tree (DT), Random Forest (RF), Logistic Regression (LR) Artificial Neural Network (ANN), Support Vector Machine (SVM) and K-Nearest Neighbor (KNN) and Multi-Layer Perceptron (MLP).

The training method used was K-Fold cross-validation with K set to 10. Therefore, the results obtained are more reliable than using the Train Test Split method. In this case, the data set was divided into 10 random subsets, where one of them is used for testing and the rest for training the model. This process was repeated K times and the final performance results from the average of the values obtained during this process, considering that the main objective lies in the correct classification of samples from healthy patients and patients with pathology. Table S3 shows the results obtained for the *'C vs LC all stages'* dataset. For each classifier, the results obtained are broken down by class, as well as their average values.

It should also be worth mentioning that the lower performance of the DT and RF models could be explained not only by the high dimensionality of the data, but also by the possible data overfitting, which is a known issue when using this type of classifier.

**Table S3.** Obtained results for the 'C vs LC all stages' dataset with 95% confidence intervals.

|  | Class | Accuracy | Precision | Recall | F1 - Score | Support |
| --- | --- | --- | --- | --- | --- | --- |
| **LR** | 0 | - | 0.89 (0.86-0.92) | 0.89 (0.87-0.92) | 0.89 (0.87-0.91) | 512 |
|  | 1 | - | 0.83 (0.79-0.87) | 0.83 (0.78-0.87) | 0.83 (0.80-0.86) | 324 |
|  |  | **0.87 (0.844–0.891)** | 0.86 | 0.86 | 0.86 | 836 |
|  |  |  |  |  |  |  |
| **KNN** | 0 | - | 0.92 (0.90-0.94) | 0.92 (0.89-0.94) | 0.92 (0.90-0.94) | 512 |
|  | 1 | - | 0.87 (0.83-0.90) | 0.88 (0.84-0.91) | 0.87 (0.84-0.90) | 324 |
|  |  | **0.90 (0.880–0.920)** | 0.89 | 0.90 | 0.90 | 836 |
|  |  |  |  |  |  |  |
| **SVM** | 0 | - | 0.86 (0.83-0.89) | 0.93 (0.91-0.95) | 0.90 (0.88-0.92) | 512 |
|  | 1 | - | 0.88 (0.84-0.91) | 0.77 (0.72-0.81) | 0.82 (0.78-0.85) | 324 |
|  |  | **0.87 (0.842–0.890)** | 0.87 | 0.85 | 0.86 | 836 |
|  |  |  |  |  |  |  |
| **DT** | 0 | - | 0.84 (0.81-0.87) | 0.91 (0.88-0.93) | 0.87 (0.85-0.89) | 512 |
|  | 1 | - | 0.83 (0.79-0.88) | 0.73 (0.68-0.78) | 0.78 (0.74-0.81) | 324 |
|  |  | **0.84 (0.810–0.864)** | 0.84 | 0.82 | 0.82 | 836 |
|  |  |  |  |  |  |  |
| **RF** | 0 | - | 0.87 (0.84-0.89) | 0.92 (0.89-0.94) | 0.89 (0.87-0.91) | 512 |
|  | 1 | - | 0.86 (0.82-0.89) | 0.78 (0.73-0.82) | 0.81 (0.78-0.85) | 324 |
|  |  | **0.86 (0.837–0.886)** | 0.86 | 0.85 | 0.86 | 836 |
|  |  |  |  |  |  |  |
| **MLP** | 0 | - | 0.90 (0.87-0.92) | 0.89 (0.87-0.92) | 0.90 (0.88-0.92) | 512 |
|  | 1 | - | 0.83 (0.80-0.87) | 0.84 (0.80-0.88) | 0.84 (0.80-0.87) | 324 |
|  |  | **0.88 (0.849–0.896)** | 0.87 | 0.87 | 0.87 | 836 |

Analyzing the results obtained, it appears that models that somehow use distance calculation between samples (KNN) or models that use linear equations (SVM and LR) presented better performance for the proposed problem, which may suggest that perhaps there is a natural 'structure' of the data capable of distinguishing samples from healthy patients from patients with pathology. It is noteworthy that the difficulty in classifying samples from LC patients with pathology can be explained by the existence of a lower number of samples of this type, when compared to samples from healthy patients (292 vs 492). In other words, the data imbalance between classes is a factor that hinders the classification of LC patient’s samples.

LR proved to be a very robust model, capable of effectively classifying samples from both classes. Although it outperformed for class 0, with high precision (0.89) and recall (0.89) respectively, for class 1 it had slightly lower but equally positive values (0.83 for both parameters, respectively) in general terms.

KNN performed strongly for both classes. For class 0, it obtained a high precision (0.92) and recall (0.92), respectively, and for class 1, also very high values (0.87 and 0.88 by the same order) with no difficulty in classifying patients with pathology. The robustness and high performance of this model could be confirmed by the high F1-Score (0.90).

The SVM showed a very positive overall performance (0.87), with the precision and recall indicators performing well for both classes (0.86 and 0.93, respectively) and (0.88 and 0.77 by the same order), respectively. It also has a high F1-Score (0.86). Overall, it proved to be a very robust and reliable model for classifying different samples.

DT displayed a high recall for class 0 (0.91) but had some difficulty classifying patients with pathology (0.73). However, the classification given was usually a correct classification since we found a very acceptable precision (0.84). Although the overall performance (0.84) was good, it showed some room for improvement.

In RF there was an improvement in the classification of patients with pathology, where we had a higher recall (0.77) and a similar overall performance (0.86) when compared to the DT method. In general, and because it is a method made up of several decision trees, it had a more robust F1-Score (0.85), which indicated better generalization in the classification of new data compared to the DT method used solely.

The Multi-Layer Perceptron (MLP) is an Artificial Neural Network (ANN). This type of classifier is known for its versatility and extraordinary ability to understand data and deliver solid results. Although there was a slight difference between classes, the results were very encouraging, and the model proved to be effective in classifying the different samples. For class 0, both precision (0.90) and recall (0.90) were very high showing a strong performance when it came to classifying healthy patients. For class 1, despite a slightly lower performance (0.85 for both parameters), the results were good and showed that the model could effectively classify patients with pathologies. This could also be confirmed by the high value of the F1-Score (0.88).

The analysis carried out on the *'C vs LC all stages'* dataset was mainly aimed at distinguishing between samples of healthy patients and patients with pathologies. Similarly, it was hoped that this would also be possible for the *'C vs LC stage IA'* dataset. The results obtained are summarized in Table S4.

**Table S4.** Obtained results for the 'C vs LC stage IA' dataset with 95% confidence intervals.

|  | Class | Accuracy | Precision | Recall | F1 - Score | Support |
| --- | --- | --- | --- | --- | --- | --- |
| **LR** | 0 | - | 0.92 (0.89-0.94) | 0.96 (0.94-0.97) | 0.94 (0.92-0.95) | 492 |
|  | 1 | - | 0.80 (0.72-0.87) | 0.66 (0.58-0.74) | 0.72 (0.66-0.79) | 128 |
|  |  | **0.90 (0.874–0.921)** | 0.86 | 0.81 | 0.83 | 620 |
|  |  |  |  |  |  |  |
| **KNN** | 0 | - | 0.93 (0.90-0.95) | 0.96 (0.94-0.97) | 0.94 (0.93-0.95) | 492 |
|  | 1 | - | 0.80 (0.72-0.87) | 0.70 (0.62-0.78) | 0.75 (0.68-0.80) | 128 |
|  |  | **0.90 (0.880–0.927)** | 0.86 | 0.83 | 0.84 | 620 |
|  |  |  |  |  |  |  |
| **SVM** | 0 | - | 0.90 (0.87-0.92) | 0.98 (0.96-0.99) | 0.94 (0.92-0.95) | 492 |
|  | 1 | - | 0.87 (0.79-0.93) | 0.57 (0.48-0.65) | 0.69 (0.61-0.76) | 128 |
|  |  | **0.89 (0.869–0.918)** | 0.88 | 0.77 | 0.81 | 620 |
|  |  |  |  |  |  |  |
| **DT** | 0 | - | 0.87 (0.84-0.90) | 0.95 (0.93-0.97) | 0.91 (0.89-0.93) | 492 |
|  | 1 | - | 0.70 (0.60-0.79) | 0.47 (0.39-0.56) | 0.56 (0.48-0.64) | 128 |
|  |  | **0.85 (0.821–0.879)** | 0.79 | 0.71 | 0.74 | 620 |
|  |  |  |  |  |  |  |
| **RF** | 0 | - | 0.90 (0.86-0.91) | 0.98 (0.96-0.99) | 0.93 (0.91-0.95) | 492 |
|  | 1 | - | 0.87 (0.78-0.93) | 0.56 (0.43-0.61) | 0.68 (0.56-0.72) | 128 |
|  |  | **0.89 (0.868–0.914)** | 0.88 | 0.77 | 0.81 | 620 |
|  |  |  |  |  |  |  |
| **MLP** | 0 | - | 0.94 (0.91-0.96) | 0.93 (0.91-0.95) | 0.93 (0.92-0.95) | 492 |
|  | 1 | - | 0.74 (0.66-0.81) | 0.76 (0.68-0.83) | 0.75 (0.69-0.80) | 128 |
|  |  | **0.91 (0.890–0.932)** | 0.87 | 0.86 | 0.86 | 620 |

Given the quality of the results obtained for the previous dataset, the same approach and models were used to classify the samples present in the *'C vs LC stage IA'* dataset. As with the previous results, most of the classifiers performed between 85 and 90% correctly (Tables S3 and S4). The models were highly capable of correctly classifying samples from healthy patients and found it more difficult to classify samples from patients with pathologies, as was also seen previously. This difference was largely due to the imbalance in the number of samples (492 vs. 128), which was more evident in the performance of some classifiers. One more time, the dataset’s classes distribution is evidence of the noticeable imbalance that influences the classification of the lung cancer stage IA group.

LR proved to be a model capable of effectively classifying samples from both classes. Although it performed much better for class 0, with high precision (0.92) and recall (0.96) respectively, for class 1 it had slightly more modest but equally positive values (0.80 and 0.66 for the same parameters, respectively) when compared to the other classifiers.

KNN performed strongly for both classes. For class 0, it obtained high precision (0.93) and recall (0.96) marks, respectively, and for class 1, also high-quality values (0.80 and 0.70 for the same parameters in the same order), although it had some difficulty in classifying patients with pathology. The robustness of this model can be confirmed by the good value obtained for F1-Score (0.84).

The SVM algorithm showed a very positive overall performance (0.89), with the precision (0.90) and recall (0.98) indicators performing very well for class 0, but falling a little short for class 1, especially regarding recall (0.57). It also had a good F1-Score (0.81).

DT showed a high recall for class 0 (0.95) but had difficulties in classifying patients with pathology (0.47). Although the overall performance (0.85) was good, there is some room for improvement.

In RF there was an improvement in the classification of patients with pathology, where we had a slightly higher recall (0.56) and a slightly better overall performance (0.89) when compared to DT. In general, and because it is a method made up of several decision trees, as mentioned before, it had a more robust F1-Score (0.84 vs 0.74) than the DT method alone.

The Multi-Layer Perceptron (MLP) neural network once again stood out for its extraordinary ability to understand the data and present solid results compared to the other classifiers. Although there was still a difference between classes, the results were very encouraging, and the model proved to be effective in classifying the different samples. For class 0, both precision (0.94) and recall (0.95) were excellent, showing a strong performance when it came to classifying healthy patients. For class 1, despite a slightly lower performance (0.80 and 0.77 for the same parameters, respectively), the results were higher than those obtained by the other models and showed that it could effectively classify patients with pathologies. This was also confirmed by the F1-Score value (0.86), the highest of all the models.

In addition, these algorithms were used to analyze whether adenocarcinomas could be detected independently of any other histological types. The results are shown in Table S5. In this case, the slight degradation of the performance indicators was mostly related to the dramatic decrease in the number of samples of the universe used for the classification.

**Table S5.** Obtained results for the 'Adenocarcinoma vs. Other Histology ' dataset.

|  | Class | Accuracy | Precision | Recall | F1 - Score | Support |
| --- | --- | --- | --- | --- | --- | --- |
| **KNN** | 0 | - | 0.75 | 0.72 | 0.73 | 196 |
|  | 1 | - | 0.75 | 0.79 | 0.77 | 96 |
|  |  | **0.75** | 0.75 | 0.75 | 0.75 | 292 |
|  |  |  |  |  |  |  |
| **SVM** | 0 | - | 0.72 | 0.65 | 0.68 | 196 |
|  | 1 | - | 0.71 | 0.77 | 0.74 | 96 |
|  |  | **0.71** | 0.71 | 0.71 | 0.71 | 292 |
|  |  |  |  |  |  |  |
| **MLP** | 0 | - | 0.75 | 0.72 | 0.74 | 196 |
|  | 1 | - | 0.75 | 0.79 | 0.77 | 96 |
|  |  | **0.75** | 0.75 | 0.85 | 0.75 | 292 |
